# Extracts and Fractions from Edible Roots of *Sechium edule* (Jacq.) Sw. with Antihypertensive Activity

**DOI:** 10.1155/2014/594326

**Published:** 2014-04-09

**Authors:** Galia Lombardo-Earl, Rubén Roman-Ramos, Alejandro Zamilpa, Maribel Herrera-Ruiz, Gabriela Rosas-Salgado, Jaime Tortoriello, Enrique Jiménez-Ferrer

**Affiliations:** ^1^Programa de Doctorado en Ciencias Biológicas y de la Salud, División de Ciencias Biológicas y de la Salud, Universidad Autónoma Metropolitana Iztapalapa, 09340 México, DF, Mexico; ^2^Centro de Investigación Biomédica del Sur (CIBIS-IMSS), Argentina 1, Colonia Centro, 62790 Xochitepec, MOR, Mexico; ^3^Facultad de Medicina, Universidad Autónoma del Estado de Morelos, Calle Iztaccihuátl Esq. Leñeros S/N, Colonia Volcanes, 62350 Cuernavaca, MOR, Mexico

## Abstract

*Sechium edule* is traditionally used in Mexico as a therapeutic resource against renal diseases and to control high blood pressure. The purpose of this work is to evaluate the antihypertensive effect of the hydroalcoholic extract obtained from the roots of this plant, including its fractions and subfractions, on different hypertension models induced with angiotensin II (AG II). The hydroalcoholic extract was tested on an *in vitro* study of isolated aorta rings denuded of endothelial cells, using AG II as the agonist; this assay proved the vasorelaxant effect of this extract. Vagotomized rats were administered different doses of AG II as well as the Hydroalcoholic extract, which reduced blood pressure in 30 mmHg approximately; subsequently this extract was separated into two fractions (acetone and methanol) which were evaluated in the acute hypertension mouse model induced with AG II, where the acetone fraction was identified as the most effective one and was subsequently subfractioned using an open chromatographic column packed with silica gel. The subfractions were also evaluated in the acute hypertension model. Finally, the extract, fraction, and active subfraction were analyzed by MS-PDA-HPLC, identifying cinnamic derivative compounds like cinnamic acid methyl ester.

## 1. Introduction

*Sechium edule* (Jacq.) Sw. (Cucurbitaceae) is an endemic plant from Mexico known as chayote. It is originally from southern Mexico (states of Veracruz, Puebla, and Oaxaca) and was domesticated in the valleys of Oaxaca and Tehuacan, Puebla [1].* S. edule* is cultivated for alimentary purposes, where the stems and fruits are mainly used, although the roots are specially appreciated in the east and southeast of Mexico [2]. The roots are described as a succulent fibrous tuber with a characteristic flavour [3]. Also, this plant is used in the Mexican traditional medicine; in Motozintla, Chiapas, the “Mam” indigenous community employs the chayote plant for treating symptoms such as severe headaches with ringing ears, nervousness, and anxiety, where a decoction of the leaves is prepared for use as drinking water throughout the day [4]. Its use is also reported for the treatment of renal and urinary disorders, like bladder or kidney stones, inflammation of the urethra, and difficulty and pain when urinating, as well as high blood pressure, varicose veins, and venous insufficiency, among others [5, 6].

There has been little pharmacological research done on* S. edule*. These studies have reported the hypotensive activity of aqueous and hydroalcoholic extracts from aerial parts of the plant; the treatments were administered orally and intraperitoneally in rats and dogs, respectively [7, 8]. Other reports have indicated that the hydroalcoholic extract of fruits of two varieties of* S. edule,* which were administered intravenously in anaesthetized rats, produced effects on different cardiovascular parameters such as lowering blood pressure and displaying changes on electrocardiographic (ECG) recordings [9]. Nonetheless, the action mechanisms by which these extracts act are not described. Phytochemical analysis has been done on the hydroalcoholic extracts of roots and aerial parts of* S. edule* plants collected in Italy, where chemical differences between the two plant organs were reported. The aerial parts contained a high concentration of luteolin glycosides, while the most significant concentration of apigenin derivatives (C-glycosidic and O-glycosidic bonds) was found in the root extract [10]. The aim of the current study was to evaluate the antihypertensive effect of* S. edule* root extract and fractions on two different murine models, in which the participation of the extracts as a calcium antagonist is proposed, as well as the possible modulation of AG II, together with the chemical identification of the active compounds implicated in the pharmacological activity.

## 2. Materials and Methods

### 2.1. Plant Material and Extract Preparation

*Sechium edule* (Jacq.) Sw. roots and aerial parts were collected in the community of Tuxpanguillo, Veracruz, Mexico (18°47′00.5′′ N and 97°00′17.5′′W, 1721 meters above mean sea level), during the months of April and May. Plant material was identified by Abigail Aguilar-Contreras, M.S., and the IMSSM Herbarium Director (located in National Medical Center, Mexico City). Voucher specimens were stored for future reference (IMSSM-15549). The roots (R) were dehydrated by freeze-drying and ground in an electric mill (Pulvex) obtaining particles of <4 mm. An exhaustive maceration was conducted with ethanol/water (60 : 40) mix at room temperature, concentrated by evaporation at reduced pressure and controlled temperature, and finally dried by lyophilization, obtaining a hydroalcoholic root extract (SeRHA). After a pharmacological evaluation of this extract on vagotomized rats, it was submitted to a prechemical separation with acetone following with methanol, obtaining the acetone extract (SeRAce) and the methanol extract (SeRMeOH).

Based on the results obtained from the acute pharmacological evaluation, SeRAce (2 g) was then subjected to chemical separation by a chromatographic column (64 × 5 cm) packed with Silica-Gel 60 Merck (0040–0063 mm). The column was eluted against a gradient of chloroform : methanol. Forty-one fractions were obtained, which then were analysed by thin layer chromatography (silica gel on aluminium sheets 60 F245 Merck). Based on their chromatographic profile, 20 fractions were grouped and named R1 to R20; from these fractions, the eight most representative ones were evaluated on the acute hypertension mice model and two were characterized by HPLC, using HPLC module system (Waters 2595) coupled with a PDA detector (Waters 996) and Empower Chromatographic Manager software version 1 empower pro (Waters). The stationary phase corresponded to a column Altima HP C18-HL Rocket (53 × 7 mm; 3 *μ*m). The mobile phase consisted of a gradient (Table 1) of 0.5% TFA : CH_3_CN 1 : 1 at a rate flow of 1 mL/min for 24 min; using a photodiode detector array, the wavelengths were scanned at 250–600 nm and the main compounds were analyzed at 340 nm for flavonoids and at 290 nm for cinnamic derivatives.

The extracts and fractions were also analysed by MS-PDA-HPLC to identify the molecular weight of the active compounds. The method used was as follows: for the HPLC analysis, liquid chromatographer (Agilent 1200) with a visible-UV diode array detector (Waters model 2996) was used, with a column Zorbax Eclipse Plus C18 100 × 2.1 mm, 3.5 *μ*m. The eluent used was acetonitrile and water/0.2% acetic acid, starting at 10 : 90 and at 30 min 90 : 10, respectively. The temperature for the column was 40°C with a flow rate of 0.2 mL/min; the results were read at 300 nm. MS was performed on an Esquire 6000, operating with negative ion polarity, ESI ionization mode, and negative current alternating ion.

### 2.2. Animals

Male Sprague-Dawley albino rats (250–280 g) and male ICR albino mice (30–36 g) from Harlan, Mexico City, were used. All animals were housed (8 per cage) and maintained under laboratory conditions: 25°C, a normal 12 h : 12 h light/dark schedule (lights on at 07:00 a.m.) with free access to water and food (pellets from Harlan rodent lab diet). The animals were given at least three weeks to adapt to the laboratory environment before experiments. All studies were carried out in accordance with the official Mexican regulation NOM-062-ZOO-1999 (technical specifications for production, care, and use of laboratory animals); the experimental protocol including the ethical aspects was authorized since December 14, 2010, by the Local Health Research Committee (IMSS, Registry number: R-2010-1701-62).

### 2.3. Angiotensin II Induced Hypertension in Vagotomized Rats

Rats were put under surgical anaesthesia with urethane (1.5 g/kg,* i.p*.), the cervical vagus nerve was cut, and the femoral vein was dissected in order to allow drug administration. Blood pressure (BP) was measured thought indirect blood pressure equipment (LE 5002 Storage Pressure Meter, Biopac Systems MP 150). In all animals, hexamethonium chloride (ganglion-blocking agent) was administered at 0.1 g/kg* i.v*. dose. After BP stabilization, increasing dosages of AG II (0.5, 1, and 2 *μ*g/kg) were administered to establish a dose dependent curve. BP was recorded every 20 s for 8 min following each dose. After recording the baseline blood pressure, three doses of SeRHA were administered (50, 100, and 200 mg/kg* o.p*.). One hour later, BP was measured for a period of 5 min; immediately, AG II was injected* i.v.,* at the doses previously given, and BP was measured again.

### 2.4. Acute Hypertension Induced on Mice with AG II

Based on the acute high blood pressure model proposed by Jiménez-Ferrer et al. (2010) [11] to establish an acute model of hypertension on mice, it was necessary to determine which dose of AG II was enough to elevate SBP and DBP over the normal parameters (120/70 mmHg). A dose dependent curve was done (data not shown). The optimal dose defined was 1 *μ*g/kg of AG II (Sigma) by* i.v. *administration. Subsequently a bioguided analysis was performed to define which extract (SeRHA, SeRAce, or SeRMeOH, 50 mg/kg,* o.p*.) exhibited a higher antihypertensive activity. The three extracts were administered by* o.p.,* one hour prior to the trial, as well as Losartan (10 mg/kg,* o.p.*) an AT_1_ receptor antagonist of AG II that was used as the positive control. The experiment control group received isotonic saline solution (100 *μ*L/10 g,* o.p.*). One hour after the treatments, under surgical anaesthesia (pentobarbital 55 *μ*g/kg,* i.p.*), BP was monitored by a noninvasive BP detector (LE 5002 Storage Pressure Meter, Biopac Systems MP150); 8 BP lectures were taken to establish a base line, immediately after AG II was administered and the BP was registered again. At this point, the SeRAce extract was chosen for the chemical separation by chromatographic column. Representative fractions (R2, R5, R6, R8, R11, R14, R17, and R20) were also tested in this same model.

### 2.5. *In Vitro* Aorta Ring Assay

Rats were sacrificed by decapitation, the thoracic aorta was isolated, cleaned of fat and connective tissue, and all aortas were denuded of endothelium film by gentle mechanical procedure and, finally, cut into rings of about 4-5 mm of width. The rings were tied to stainless steel hooks with silk thread and immersed into 10 mL organ baths of Krebs solution at 37°C and oxygenated (O_2_/CO_2_, 95 : 5). A basal tension of 2 g was established for all tissues, and Biopac Systems TSD 125c equipped with AcqKnowledge software recorded changes in basal tension. KCl (120 mM) was administered to determine the maximum contraction of each prepared aorta.

Sixty min after stabilizing the tissue, rings were stimulated with different concentrations of AG II (from 1.00 × 10^−10^ to 1.00 × 10^−6^ M) for 10 min and then washed out to remove stimulant agent and stabilized for another 30 min. The tissues were preincubated with SeRHA (150 *μ*g/mL, 300 *μ*g/mL, and 600 *μ*g/mL), respectively. The relaxant effect was determined by comparison among maximum vascular contraction before and after addition of samples.

To determine the possible vasorelaxant mechanism (like calcium antagonism), It was evaluate the effect of calcium over AG II agonistic activity, the assay consisted of evaluating a calcium concentration curve inducing vasoconstriction with AG II at a specific concentration (1 × 10^−6^ M) and the activity of SeRHA (200 *μ*g/mL, 400 *μ*g/mL, and 800 *μ*g/mL); Nifedipine (calcium channel blocker) was employed as positive control.

### 2.6. Statistical Analysis

Statistical analysis was carried out with SPSS 11.0 and based on an analysis of variance (ANOVA) followed by Bonferroni test. A significant difference was established with respect to control group when the *P* value was lower than 0.05.

## 3. Results 

### 3.1. Effect of* S. edule* Root Extract on Vagotomized Rats with AG II Induced Hypertension

Blood pressure elevation on vagotomized rats was established by a dose dependent curve, as showed in Table 2.

**Figure 1:**
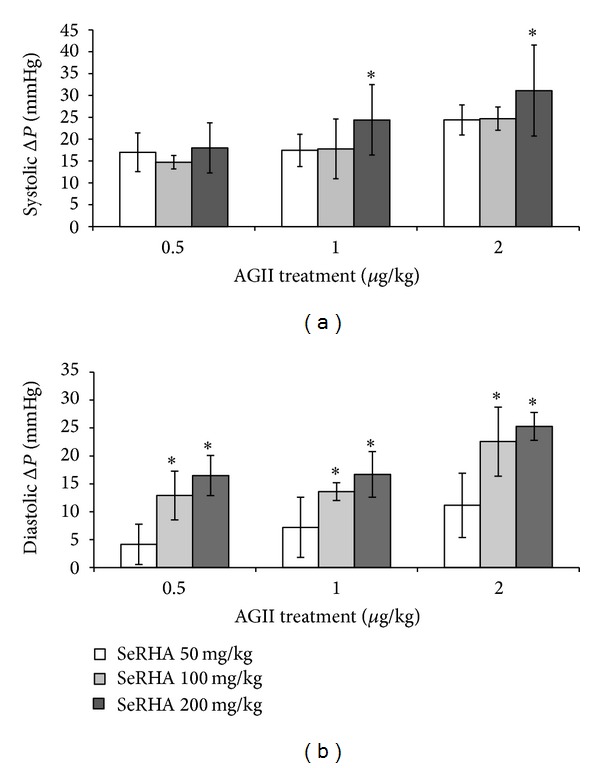
Antihypertensive activity of increasing doses (50, 100, and 200 mg/kg* i.p.*) of SeRHA, in vagotomized rats with ganglion blockage by hexamethonium chloride, pretreated with AG II* i.v*. Each bar represents the average of the differential of systolic (a) and diastolic (b) blood pressure with respect to the basal pressure, after AG II administration. ANOVA,* post hoc* Bonferroni  *P < 0.05, when compared to SeRHA 50 mg/kg represented in white bars (*n* = 6; mean ± SE).

The administration of the different dosages of SeRHA extract was capable of inhibiting the increment of BP when given the respective dose of AG II, the effect of which was preestablished in the dose dependent curve.  Figure 1 indicates the differential of the decrement of the systolic blood pressure (SBP) and diastolic blood pressure (DBP) when given AG II. Once the highest dose of AG II (2.0 *μ*g/kg) is administered in all three SeRHA treatments, there is a proportional drop in BP. In Figure 1(a) the 200 mg/kg dose of SeRHA showed a statistical difference when the 2.0 *μ*g/kg AG II dose was administered, decreasing BP in 30 mmHg (*F*(2.93) < 53.06, *P* < 0.05) when compared with differential effect (de) induced with the lower dose of AG II (0.5 *μ*g/kg) and SeRHA (50 mg/kg). The behaviour of the hypotensive activity from the SeRHA 100 and 200 mg/kg dose was proportional to the administration of the AG II dose, making it a dose dependent drop in SBP, where BP dropped 24 mmHg, with a statistical difference of both groups of *F*(2.93) < 152.96, *P* < 0.05, when compared with differential effect (de) induced with the lower dose of AG II (0.5 *μ*g/kg) and SeRHA (50 mg/kg). This activity could be consistent with the modification of the cardiac output of the animals and this might be the cause why Lozoya et al. [7, 8] saw a modification on the ECG recordings when they tested the fruit and leaf extracts.

The modification of DBP is shown in Figure 1(b), where the three dosages of SeRHA produced an important effect preventing an increase in DBP when administered the different AG II doses. These results were more evident than those of SBP, since only the 100 and 200 mg/kg doses of SeRHA that were administered in all three AG II groups showed the most important hypotensive activity compared with the smallest dose of SeRHA and AG II that are exhibited as the white bars, as well as in the SBP results; the highest dose of AG II and of SeRHA had the most potent effect, with a fall of DBP of around 25 mmHg. The impairment of the increment of DBP by the SeRHA extract in this model might be associated with an AG II antagonist activity, due to the nature of this model, where one of the ways to block AG II effect would be the extract interacting with the AT_1_ receptor for AG II, since the vagotomization and ganglionic blockage inhibit the BP control mechanisms in general, and the model is induced by intravenous injection of the agonistic AG II, leaving this mechanism of action as the most prominent to occur.

### 3.2. Ring Aorta Assay

**Figure 2:**
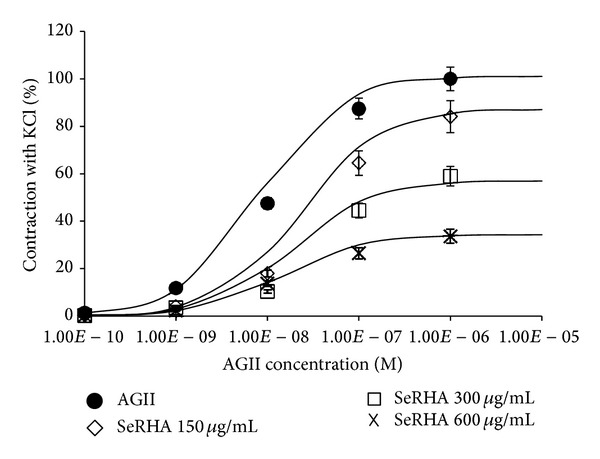
Percentages of the contraction of vascular smooth muscle of rat aorta rings stimulated with increasing concentrations of AG II, with respect to the maximum effect obtained with KCl, as well as the evaluation of the vasorelaxant effect of the SeRHA extract stimulated with AG II.

**Figure 3:**
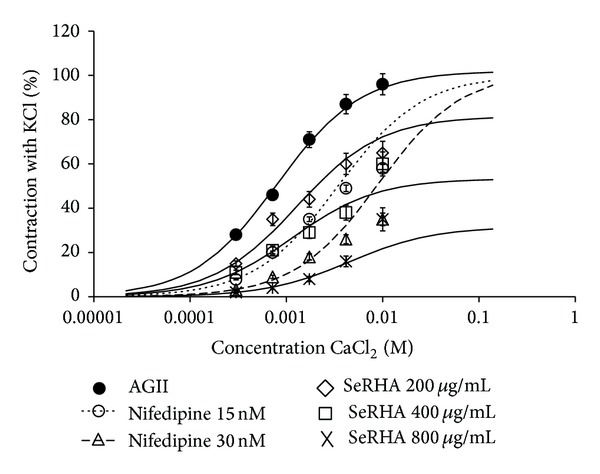
Percentages of the contraction of vascular smooth muscle of rat aorta rings stimulated with AG II, in presence of increasing concentrations of CaCl_2 _with respect to the maximum effect obtained with KCl, as well as the modulating effect of SeRHA extract on the contraction. Nifedipine was used as a positive control due to its calcium channel blocker activity.

Figures 2 and 3 show the behaviour of the isolated rat aortas without endothelial cells, under two conditions of stimulation of vascular smooth muscle. The resulting contraction is reported with regard to the maximum contraction produced by the administration of KCl.

Figure 2 presents the aorta contraction curve dependent on the increasing concentration of AG II (∙), with a *E*_max⁡_ = 98% and a EC_50_ = 8.5 × 10^−9^ M. It also shows the effect of three doses of SeRHA + AG II, 150 *μ*g/mL (⋄), 300 *μ*g/mL (□), and 600 *μ*g/mL (×); all three extracts had a statistical difference with respect to AG II, where the first concentration of SeRHA had a decrease of 14% of *E*_max⁡_, the second one of 44% of *E*_max⁡_, and the highest concentration diminished the contraction 66% of *E*_max⁡_. The EC_50_ values for the three concentrations of SeRHA were with an average of 1.5 × 10^−8^ M.

Figure 3 shows the Ca^++^ concentration curve, stimulated with a constant concentration of AG II (1 × 10^−6^ M) (∙). The contraction of the aorta was dependent on Ca^++^ concentration. The AG II *E*_max⁡_ value was 98% of contraction with an EC_50_ = 0.0008 M. When SeRHA + AG II was administered, the resulting effect had a statistically significant decrease of *E*_max⁡_ where SeRHA of 200 *μ*g/mL (⋄) decreased 20%, 400 *μ*g/mL (□) decreased 48%, and finally the 800 *μ*g/mL (×) concentrations produced a 69% contraction of the aorta with respect to AG II by itself; the values of EC_50_ obtained were of 0.003 M in average. In this experiment, Nifedipine was used as a positive control due to its calcium channel blocker effect.

### 3.3. Bioguided Fractioning of SeRHA, Using the Acute Hypertension Mice Model Induced with AG II

**Figure 4:**
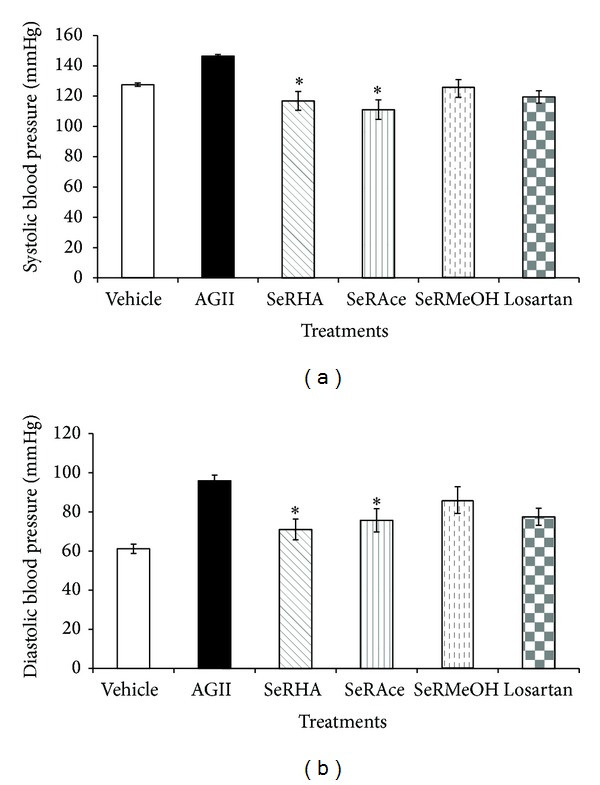
Bioguided assay of* S. edule* extracts against AG II agonistic effect. ANOVA,* post hoc* Bonferroni  *P < 0.05, when compared to the vehicle represented in white bars (*n* = 6; mean ± SE).

Having established the hypotensive activity of the SeRHA extract, it is important to identify which group of compounds is responsible for the biological activity. The extract was summited to a chemical separation, and two fractions, SeRAce and SeRMeOH extracts, were obtained. The oral administration of the extract and fractions was able to reduce the blood pressure while administering AG II (1.0 *μ*g/kg* i.v.*) (Figure 4). The most active fraction was SeRAce and the complete extract SeRHA, shown by statistical differences for SBP of *F*(2.57) < 39.69, *P* < 0.05, and for DBP of *F*(2.57) < 126.76, *P* < 0.05, when compared to the negative control condition (AG II), with a critical decrease of SBP below the base line values, was established by the vehicle (ISS) group. Both Losartan and SeRMeOH did not achieve significant differences in SBP and DBP compared with SeRHA and SeRAce. Nevertheless, they lowered the BP when compared with the AG II group.

#### 3.3.1. Antihypertensive Activity of SeRAce Fractions

Based on the pharmacological activity of SeRAce extract, it was selected for chemical separation by chromatographic column, from which eight fractions (R2; R5; R6; R8; R11; R14; R17; and R20) were chosen based on their chromatographic profile to be evaluated on the acute hypertension mice model (Table 3). Five of the eight fractions presented a decrease in BP when administered AG II; all five fractions had a significant hypotensive activity on SBP although not all of them had the same activity on DBP. Only two fractions (R14 and R17) were able to significantly decrease SBP (*F*(2.11) < 92.92; *P* < 0.05) and DBP (*F*(2.11) < 41.18; *P* < 0.05), with a net effect below the SBP of the ISS group and of the control group Losartan. The hypotensive activity of these extracts and fractions could be related to the phenolic compounds that were identified in the chemical characterization done through HPLC of the R14 and R17 fractions and SeRHA as well as for SeRAce extracts.

### 3.4. Chemical Analysis

SeRHA, SeRAce, and the most active fractions (R14 and R17) were analysed by HPLC and MS-PDA-HPLC; the compounds in each one displayed diverse UV spectra that can be related to flavones (*λ*_max⁡_ = 340 nm) and cinnamic acid derivatives (*λ*_max⁡_ = 290 nm).

The extraction made with acetone from SeRHA allowed us to obtain a less polar fraction that also presented the antihypertensive activity that was tested in the biological model used.

**Figure 5:**
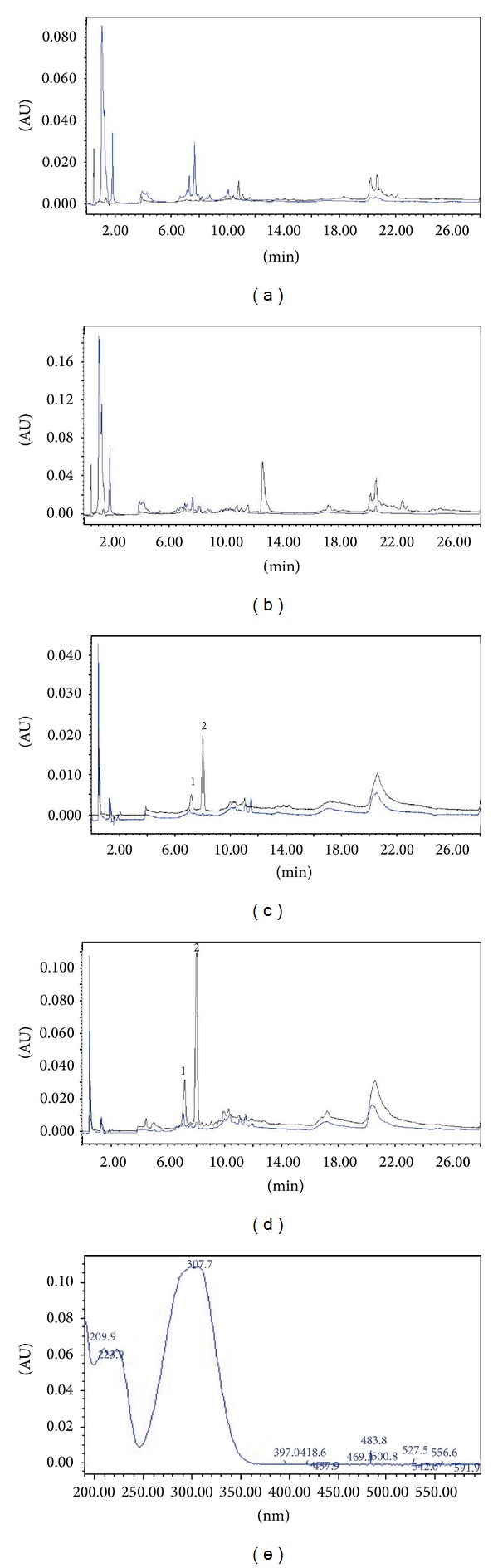
Chromatograms of SeRHA extract (blue line) compared with SeRAcet (black line); they were read at two length waves, panel (a) at 290 nm and panel (b) at 340 nm. Fractions R14 (black line) and R17 (blue line) represented in panels (c) and (d) read at 340 nm and 290 nm, respectively, show two peaks (1 and 2) with retention times of 7.21 and 8.04 min. Peak 2 corresponds to the cinnamic acid methyl ester that corresponds to the MS-PDA-HPLC analysis represented in Figure 6(a) peak 4 and Figure 6(b) peak 160.3.

**Figure 6:**
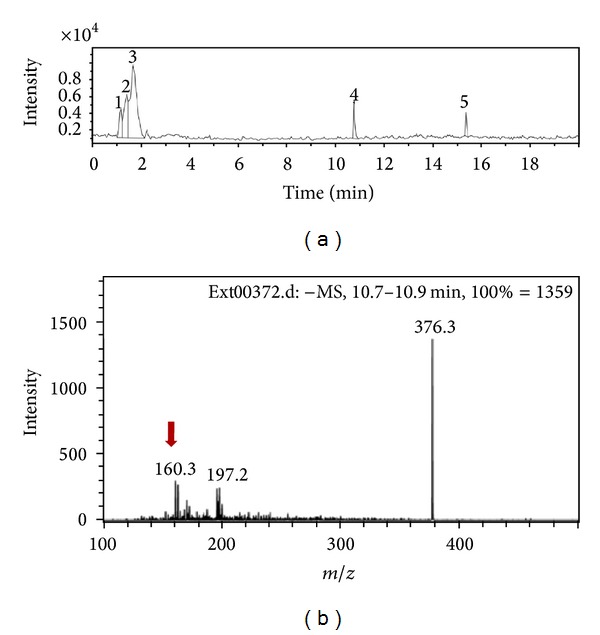
Mass chromatogram of fraction R14 (a) and its mass absorption spectrum (b).

The chromatographic comparison of SeRHA extract with the fraction SeRAcet that was read at 340 nm (specific for vitexin and other related flavonoids) (Figure 5(a)) allows observing the loss of this type of compounds in the fraction SeRAcet as well as the more polar compounds that came out with the solvent front. When the comparison of these two was analysed at 290 nm (Figure 5(b)), It can be appreciated an increment in the concentration of the peaks present throughout minute 7 to 18 of the retention times of this fraction. The chemical separation of these fraction derived 20 fractions, from which two of them, R14 and R17 (at a dose of 50 mg/kg), presented significant statistical difference in the biological activity superior to Losartan. These fractions were compared in the chromatographic method at 290 nm, observing an increment in the peaks with retention time 7.21 and 8.04 min in both fractions having the same UV spectrum for both retention times, peak 1 and 2 from Figures 5(c) and 5(d), respectively. The MS-PDA-HPLC analysis from SeRHA and the Fraction 14 (Figure 6(a)) allowed establishing that the peak with retention time of 10.8 min corresponds to a cinnamic acid methyl ester.

## 4. Discussion 

Blood pressure is a mechanism that an organism has to maintain a constant irrigation of blood, for nutrient distribution and oxygenation of organs and tissues. It is regulated essentially throughout two factors, cardiac output (CO) and total peripheral resistance (TPR). The CO is the total amount of blood pumped in to the aorta each minute by the heart and the TPR or vascular resistance is the impediment to blood flow in a vessel. The pharmacological model used in this work has the purpose of simulating an alteration in the TPR, increasing blood pressure by the administration of AG II, and triggering a general vasoconstriction reaction. Drug discovery [12] describes this assay as an AG II antagonism model.

The vagotomization of the rats eliminates the cardiovascular reflexes, without interfering with the heart rate and normal blood pressure, allowing AG II to induce an increase of blood pressure, inciting an acute vasoconstriction when administered intravenously without any intervention of the sympathetic and parasympathetic response, which are responsible for the immediate homeostatic control of blood pressure. The ganglionic-blockade purpose is to block the transmission of nerve impulses through the autonomic ganglia [13], impairing the vasculature to compensate blood pressure when administered AG II or the extract evaluated.

The results obtained from this model allowed us not only to confirm the antihypertensive effect of this plant but also to identify the probable action mechanism. The extracts of* S. edule* may have an AG II antagonism activity. This action mechanism can be modulated through direct antagonism of the AG II receptor AT_1_ or by the intervention on calcium fluxes activated by AG II, impeding the immediate vasoconstriction response to the* i.v.* administration of AG II.

In the isolated aorta assay AG II vasoconstriction action was dependent on Ca^++^ concentration, where it was observed that the increment in Ca^++^ promoted a rise of the contraction of the aorta when a single concentration of AG II was administered. Nifedipine was used as a pharmacological control; it specifically blocks L-type calcium channels as a competitive antagonist [14] on the calcium influx that can be through depolarization or by receptor-response coupling [15], which, in this assay, was triggered by AG II. The SeRHA extract, in comparison to Nifedipine, responded as an irreversible antagonist (also called pharmacologic antagonist) that shifts the dose-response curve downward, indicating that the agonist can no longer exert maximal effect at any dose. All three concentrations of the extract that were tested not only inhibited the aorta contraction but also altered the EC_50_ as a competitive antagonist would do [16].

This behaviour confirms the AG II antagonist effect, although it could also have a calcium antagonist effect. The activity of SeRHA extract can be associated with an AG II receptor blocking action, as well as obstructing the second messenger system initiated by AG II, which promotes the efflux of sarcoplasmic calcium that activates the store operated channels (SOC) that allows more calcium to enter the cell and form a calcium-calmodulin complex That ends in vascular contraction [17, 18].

With these results, the antihypertensive and vasorelaxant activity of* S. edule* root extract were demonstrated; therefore, a chemical separation was carried out, obtaining a first fraction SeRAce, which was summited to chemical separation and the most chemically relevant fractions obtained were analysed on the acute hypertensive model; two of them had the most pharmacological activity (R14 and R17) and were then analysed by HPLC as well as SeRHA and SeRAce. The chemical results allowed us to identify most polyphenolic compounds like flavonoids and phenylpropanoids. Vitexin was identified in SeRHA and SeRAce as well as coumaric and cinnamic acid; in contrast with the extracts, fractions R14 and R17 predominantly had coumaric and cinnamic acid. This information is relevant and consistent to the results obtained from the pharmacological experiments.

Polyphenolic compounds have a very wide and important biological activity regarding cardiovascular diseases. Mainly in the regulation of high blood pressure, recent studies are evoked on inflammation and ROS scavenging properties of this type of compounds [19–22]. Thus, there have been studies on flavonoids and cinnamic acid derivative on their calcium antagonism and vasorelaxant activity [23, 24].

Liew et al. [25] demonstrated that red wine polyphenols like resveratrol act as calcium channel antagonists lowering free intracellular Ca^++^. Summanen et al. [23] presented that numerous simple phenolic compounds like cinnamic acid derivatives and flavonoids have a potent inhibition of Ca^++^ entry by high K^+^-evoked activity; the effects were comparable to those of verapamil. They presented an inhibitory activity against Ca^++^ entry similar to the one of another class of natural Ca^++^ channel antagonists, like furanocoumarins identified by Vuorela et al. [26]. Other studies of extracts of* Gentiana floribunda* that contained flavonoids and tannins among other compounds were effective for lowering blood pressure with a vasorelaxant effect, mediated by the inhibition of Ca^++^ influx via membranous calcium channels and its release from the intracellular stores [27].

The presence of cinnamic acid derivatives in the* S. edule* extracts and fractions could be responsible for the angiotensin II and calcium antagonist effect, which were established with the pharmacological assays, which are designed to identify a specific action mechanism. Cinnamic acid derivatives are a group of natural compounds that have been studied in different antihypertensive models, mostly as anti-inflammatory and antioxidant activity, although these studies are based on chronic hypertension assays, which originate by the alterations of Ca^++^ fluxes in the vascular smooth muscle and on the renin—angiotensin—aldosterone system.

## 5. Conclusions 

In conclusion the results obtained in this work demonstrate that* S. edule* extracts have an antihypertensive activity, which is consistent with the traditional use in Mexico. Thus, the phytochemical analysis was not precise on a specific compound and the richness in polyphenols opens a new range of inquiry for other action mechanisms of* S. edule* extracts related to inflammation and oxidative stress damage that is related to hypertension and endothelial dysfunction.

## Figures and Tables

**Table 1:** Process conditions for HPLC chromatographic characterization of the extracts of *S. edule*.

Time (min)	Flow	0.5% TFA (%)	CH_3_CN (%)
0	1.00	100.0	0.0
1.00	1.00	100.0	0.0
2.00	1.00	90.0	10.0
4.00	1.00	90.0	10.0
5.00	1.00	80.0	20.0
7.00	1.00	80.0	20.0
8.00	1.00	70.0	30.0
14.00	1.00	70.0	30.0
15.00	1.00	60.0	40.0
18.00	1.00	60.0	40.0
19.00	1.00	20.0	80.0
22.00	1.00	20.0	80.0
23.00	1.00	0.0	100.0
26.00	1.00	0.0	100.0
27.00	1.00	100.0	0.0
28.00	1.00	100.0	0.0

**Table 2:** Effect produced on systolic and diastolic blood pressure of vagotomized rats by the administration of angiotensin II (*i.v.*).

AG II doses *μ*g/kg *i.v. *	Systolic pressure mmHg ± SD	Diastolic pressure mmHg ± S.D.
0.0	127.7 ± 3.13	55.1 ± 3.27
0.5	144.3 ± 7.34*	70.33 ± 6.15*
1.0	145.6 ± 12.2*	78.3 ± 9.30*
2.0	153.60 ± 11.0*	85.5 ± 15.4*

Data are presented as means ± SD with  *n* = 7. ANOVA and *post hoc* Bonferroni test. *P < 0.05 was compared to basal data (0.01 mg/kg AG II).

**Table 3:** Antihypertensive effect produced by losartan (10 mg/kg) and fractions (50 mg/kg) obtained from SeRAce, on pretreated mice with AG II (1.0 *μ*g/kg, *i.v. *).

Treatment	Systolic BP mmHg	Diastolic BP mmHg
Vehicle	127.51 ± 3.24	61.14 ± 6.74
AG II	146.40 ± 3.01	95.90 ± 8.01
R2	133.98 ± 15.86	86.3 ± 14.86
R5	122.61 ± 16.43	84.5 ± 18.08
R6	132.38 ± 17.87	93.03 ± 20.83
R8	138.01 ± 14.84	93.63 ± 13.98
R11	119.45 ± 19.85	82.07 ± 16.23
R14	109.21 ± 13.94*	68.88 ± 12.10*
R17	109.09 ± 8.73*	71.66 ± 14.53*
R20	122.55 ± 13.15	77.71 ± 10.97
Losartan	119.50 ± 11.73	77.45 ± 12.41

Data are presented as means ± SD with  *n* = 7. ANOVA and *post hoc* Bonferroni test. *P < 0.05 is with respect to the negative control (AG II 1.0 *μ*g/kg).
